# Intestinal NF-E2-related factor-2 expression and antioxidant activity changes in rats undergoing orthotopic liver autotransplantation

**DOI:** 10.3892/ol.2013.1576

**Published:** 2013-09-12

**Authors:** MIAN GE, XINJIN CHI, AILAN ZHANG, GANGJIAN LUO, GUOLIANG SUN, HANBIN XIE, ZIQING HEI

**Affiliations:** Department of Anesthesiology, The Third Affiliated Hospital of Sun Yat-sen University, Guangzhou, Guangdong 510630, P.R. China

**Keywords:** orthotopic liver transplantation, intestinal injury, oxidative damage, antioxidative enzyme, reactive oxygen species, NF-E2-related factor-2

## Abstract

Liver transplantation is known to trigger intestinal injuries. Oxidative damage that is induced by reactive oxygen species (ROS) plays a crucial role in ischemia-reperfusion injuries. NF-E2-related factor-2 (Nrf2) and its modulated antioxidant enzymes form the critical endogenous antioxidant system to scavenge ROS. The present study investigated the dynamic changes of intestinal ROS levels, Nrf2 expression and antioxidant enzyme activity following orthotopic liver autotransplantation (OLAT). Sprague-Dawley rats were randomly divided into five groups consisting of one sham group and four groups with rats that underwent OLAT and were evaluated following 4, 8, 16 and 24 h, respectively. The intestinal specimens were collected for histopathological examination and the detection of hydrogen peroxide (H_2_O_2_), hydroxyl radical (^•^OH), malondialdehyde (MDA), reduced glutathione (GSH), superoxide dismutase (SOD) and catalase (CAT) levels and the expression of Nrf2. The present study demonstrated that OLAT resulted in severe intestinal injury, which manifested as a significant change in the intestine pathological scores as early as 4 h and peaking at 8 h post-treatment. Oxidative stress was also revealed by the increase of the H_2_O_2_, ^•^OH and MDA levels. Significant decreases were observed in the activity of SOD and CAT and a dramatic decrease occurred in the levels of GSH at 4 and 8 h post-treatment. All the parameters were restored gradually at 16 and 24 h post-treatment. The expression of Nrf2 in the intestinal tissues increased significantly at 4, 16 and 24 h following OLAT. The present study shows that an imbalance between oxidants and antioxidants contributes to intestinal oxidative injury, and that the upregulation of Nrf2 is not sufficient to withstand intestinal oxidative injury following OLAT.

## Introduction

Post-operative multi-organ dysfunction (MODF) and systemic inflammatory reaction syndrome (SIRS) remain the main complications of liver transplantation and are critical problems that contribute to a high mortality rate (1,2). Bacterial translocation and enterogenous endotoxemia following intestinal injury may play a significant role in MODF and SIRS (3). Zheyu and Lunan (4) demonstrated that the motility and barriers of the intestine were destroyed following liver transplantation in rats, as evidenced by the increase in plasma endotoxin levels.

Liver transplantation requires the portal vein to be blocked, causing intestinal congestion, hypoxia in the anhepatic phase and the restoration of blood flow reperfusion, making intestinal ischemia-reperfusion injury inevitable (4,5). Oxidative damage induced by reactive oxygen species (ROS), including the superoxide anion (O_2_^−^), hydrogen peroxide (H_2_O_2_) and the hydroxyl radical (^•^OH), play a crucial role in the pathogenesis of ischemia-reperfusion injury (6,7). Due to the existence of antioxidative enzymes, including superoxide dismutase (SOD) and catalase (CAT), and antioxidants, including glutathione (GSH), the redox balance is well maintained and the clearance of ROS is promoted (8–10). Several studies have shown that inflammatory mediators, including tumor necrosis factor-α (TNF-α) and interleukin-1 (IL-1), are involved in the intestinal injury that is induced by liver transplantation (4,5). However, the exact alternative pattern of the oxidant/antioxidant system in the process of intestinal injury following liver transplantation remains obscure.

Nuclear factor NF-E2-related factor-2 (Nrf2) has been demonstrated to regulate the synthesis of antioxidant enzymes and to maintain the antioxidative capacity of the body (11,12). A study using microarrays revealed that Nrf2 regulated the majority of antioxidative enzyme gene expression in the lung (13). Numerous studies have reported that Nrf2 plays a pivotal role in preventing oxidative damage. However, the alterations to the expression Nrf2 remains unclear during an intestinal injury following liver transplantation.

The present study was designed to investigate the dynamic changes in the intestinal ROS levels, Nrf2 expression and antioxidant enzymes activity (SOD, CAT and GSH) occurring 24 h after orthotopic liver transplantation (OLAT), and to evaluate the imbalance of oxidants and antioxidants.

## Materials and methods

### Animals and experimental groups

This study was approved by the Institutional Animal Care and Use Committee of Sun Yat-Sen University (Guangzhou, China) and followed the National Guidelines for the treatment of animals. A total of 35 male rats weighing 220–280 g were randomly assigned into five groups consisting of one sham surgery group (sham; n=7) and four treatment groups that were tested at 4 (n=7), 8 (n=7), 16 (n=7) and 24 h (n=7) following OLAT, respectively. Subsequent to being anesthetized, the rats belonging to the sham group were subjected to a laparotomy and vascular dissection, without hepatic vascular exclusion and perfusion. The rats in the other groups underwent OLAT. The intestines, starting from 5 cm to the terminal ileum, were removed at 4, 8, 16 and 24 h following OLAT.

### Experimental model of rat OLAT

An OLAT model similar to that previously described by Jin *et al*(14) was applied with certain modifications (15). The rats were fasted for 12 h and anesthetized using inhaled ether, which was administered through an open face guard. Following the anesthesia, the abdomen was incised, the falciform ligament of the liver was resected and the blood vessels along the esophagus were ligated and disconnected. The suprahepatic vena cava (SVC) was fully liberated and the liver was replaced in its original position. Once the upper region of the left renal vein was completely free, the inferior vena cava (IVC) was dissociated. The first hepatic portal was drawn and the portal vein (PV) was separated from the splenic veins and the convergence of the inferior mesenteric vein. The hepatic artery and the biliary tract were liberated. Subsequently, the portal hepatics were ligated. Microvascular clamps were folded on the convergence of the inferior mesenteric vein, hepatic artery, splenic vein, SVC and IVC. The PV was punctured with a 4th needle in preparation for reperfusion and fixed using a microvascular clamp. Ringer lactate solution (pre-cooled, 4°C) was injected during reperfusion at a speed of 2.5 ml/min and a 1-mm incision was cut in the wall of the IVC as an outflow tract. The liver color progressively turned yellow if the reperfusion was successful. The recovery of the liver perfusion represented the accomplishment of OLAT. Finally, the needle was extracted and the incision of the PV and IVC was repaired using 8-0 sutures. The clamps on the PV, SVC, IVC and hepatic artery were loosened. The whole anhepatic phase lasted for 20±1 min.

### Histopathological examination

An intestinal segment of 0.5–1.0 cm in length was cut from 5 cm to the terminal ileum and fixed in 10% formaldehyde. Subsequent to being embedded in paraffin, the tissues were stained with hematoxylin and eosin (HE) for light microscopy examination. Intestinal mucosal damage was evaluated and graded according to the following criteria suggested by Chiu *et al*(16): Grade 0, normal mucosa villi; grade 1, development of subepithelial Gruenhagen’s space at the tip of the villi; grade 2, extension of the subepithelial space with moderate epithelial lifting; grade 3, massive epithelial lifting, possibly with a few denuded villi; grade 4, denuded villi with lamina propria and exposed capillaries; and grade 5, disintegration of the lamina propria, ulceration and hemorrhage.

### Preparation of specimens and detection of protein concentration in the small intestine

A segment of small intestine was washed with frozen saline, dried with suction paper and frozen at −80ºC. Intestinal protein levels were measured using a bicinchoninic acid assay (BCA), according to the manufacturer’s instructions (Nanjing KeyGen Biotech Co., Ltd., Nanjing, China).

### Detection of malondialdehyde (MDA) content

MDA content was measured using the thiobarbituric acid (TBA) method (Nanjing KeyGen Biotech Co., Ltd.). The homogenate (0.1 ml) was used to detect the MDA content. The condensation of MDA and TBA resulted in a red product, which had a maximum absorption peak at 532 nm. The MDA content was calculated by measuring the absorbance at 532 nm and expressed as nmol/mg protein (nmol/mg prot).

### Detection of H_2_O_2_ and ^•^OH concentrations

In the acidic environment, H_2_O_2_ is able to oxidize Fe2^+^ ions to Fe3^+^ ions, which combine with dye molecules to product Fe3^+^ dye complexes. This has a maximum absorption wavelength that was proportional to the concentration of H_2_O_2_ at 560 nm. The concentrations of H_2_O_2_ were measured by detecting the absorbance at 560 nm and were expressed as mmol/mgprot.

The levels of ^•^OH were determined through the Fenton reaction by detecting the absorbance at 550 nm. The ^•^OH level in the intestinal tissue was expressed as U/mg prot.

### Detection of SOD and CAT activities and GSH content

The intestinal tissues were homogenized with normal saline solution and centrifuged at 1,500 × g for 15 min to remove the debris. The supernatant was transferred into fresh tubes for the evaluation of the SOD and CAT activities and GSH content. The measurements were performed using an assay kit (KeyGen Biotech Co., Ltd., Nanjing, China) following the manufacturer’s instructions.

SOD activity was determined using the Xanthine oxidase method by detecting the absorbance at 550 nm. SOD activity was expressed as U/mg prot.

CAT was measured by the reaction of CAT scavenging H_2_O_2_. In this reaction, ammonium molybdate was added and a pale yellow complex was produced. The change in the absorbance at 405 nm was monitored. CAT activity was expressed as U/mg prot.

GSH reacted with dithiobis-nitrobenzoic acid to generate 5-dithio-bis2-nitrobenzoic acid dithiobis-nitrobenzoic acid anion, which was a yellow compound and had a maximum absorption peak at 420 nm. The concentrations of GSH were measured by detecting the absorbance at 420 nm and were expressed as mg/gprot.

### Western blot analysis

Nuclear proteins were extracted from the frozen intestinal tissues using protein extraction kits (KeyGen Biotech Co., Ltd.) for Nrf2 measurements. The protein concentration was measured using the Bradford method. Protein (60 μg) was loaded onto a 4–20% SDS-PAGE pre-made gel (Invitrogen, Carlsbad, CA, USA) for polyacrylamide gel electrophoresis and then transferred to a polyvinylidene fluoride membrane that was pretreated with 100% methanol. The membranes that were loaded with the protein of interest, Nrf2, were incubated with 5% skimmed milk. Rabbit monoclonal anti-Nrf2 antibody (dilution, 1:200; Santa Cruz Biotechnology, Inc., Santa Cruz, CA, USA) was added to the supernatant and the mixture was incubated on a rotating wheel at 4°C overnight. On the second day, the membranes were washed with TBS three times and incubated with a second antibody conjugated to horseradish peroxidase (dilution, 1:2,000; Santa Cruz Biotechnology, Inc.) for 1 h at room temperature. Densitometry was used, and normalization to β-actin immunoreactivity was adopted to correct the differences in the samples.

### Statistical analysis

All the data are expressed as the mean ± SD and were analyzed using SPSS 16.0 software (SPSS, Inc., Chicago, IL, USA). Repeated measurements were applied for the intra-group comparison. P<0.05 was considered to indicate a statistically significant difference.

## Results

### Intestinal pathology under light microscopy

**Figure 1:**
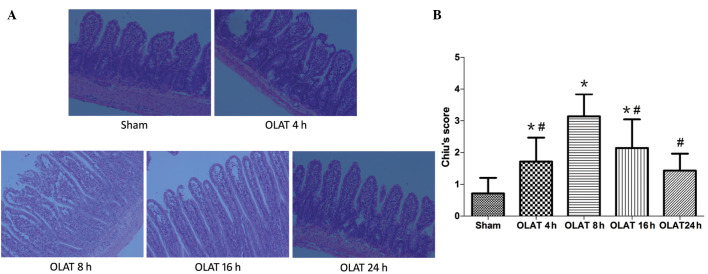
(A) Histological analysis and change in (B) Chiu’s score of small intestinal mucosa following HE staining (×200). Results are presented as the mean ± SD (n=7/group). ^*^P<0.05 compared with the sham group; ^#^P<0.05 compared with the 8-h group. OLAT, orthotopic liver autotransplantation.

No injury was evident in the sham group, which demonstrated normal mucosal villi and glands. However, in the OLAT-induced intestinal injury groups (Fig. 1), the intestinal structure was damaged most severely at 8 h following OLAT, where almost all the animals demonstrated massive epithelial lifting down the sides of the villi, with a few denuded villi. The intestinal structure was less damaged at 4 and 16 h following OLAT compared with the animals with a few denuded villi, accompanied with an extension of the sub-epithelial space with moderate or massive epithelial lifting. The Chiu’s scores significantly increased at 4, 8 and 16 h following OLAT compared with the sham group (P<0.05 vs. sham group). The Chiu’s scores peaked at 8 h (P<0.05 vs. the sham, 4-, 16- and 24-h groups) and then improved gradually (Fig. 1).

### MDA content in the small intestine

**Figure 2:**
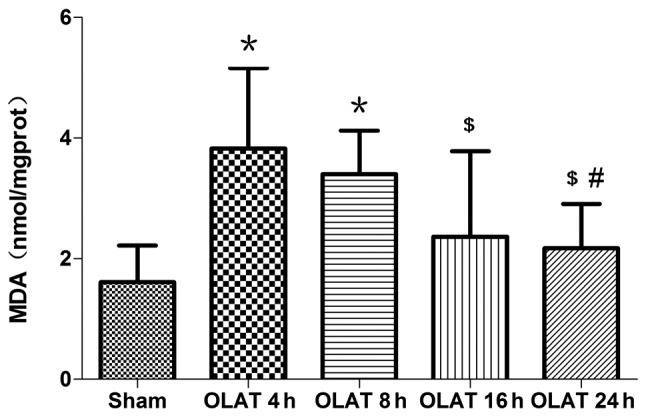
Changes of malondialdehyde (MDA) content in the small intestine Results are presented as the mean ± SD (n=7/group). ^*^P<0.05 compared with the sham group; ^$^P<0.05 compared with the 4-h group; ^#^P<0.05 compared with the 8-h group. OLAT, orthotopic liver autotransplantation.

The content of MDA increased significantly at 4 and 8 h following OLAT; the content was increased up to 2–2.4 times (P<0.05 vs. sham group). The MDA content in the 16- and 24-h groups gradually decreased (P<0.05 vs. 4-h group; Fig. 2).

### H_2_O_2_ content and ^•^OH level in the small intestine

**Figure 3:**
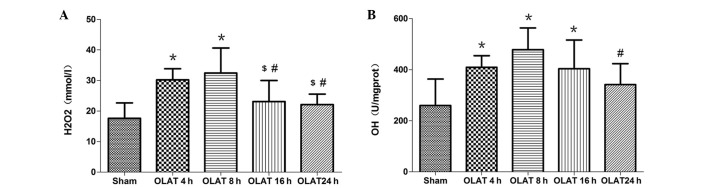
Changes in (A) H_2_O_2_ content and (B) ^•^OH levels in the small intestine. Results are expressed as the mean ± SD (n=7/group). ^*^P<0.05 compared with the sham group; ^$^P<0.05 compared with the 4-h group; ^#^P<0.05 compared with the 8-h group. OLAT, orthotopic liver autotransplantation.

The content of H_2_O_2_ increased by 1.7–1.8 times at 4 and 8 h following OLAT (P<0.05 vs. sham group; Fig. 3). The content of H_2_O_2_ gradually decreased at 16-and 24-h (P<0.05 vs. 4- and 8-h groups). Compared with the sham group, the levels of ^•^OH increased at 4, 8 and 16 h following OLAT. The increase ranged between 1.6–1.8 times (P<0.05 vs. sham group). The levels of ^•^OH at 24 h were lower than those at 8 h (P<0.05; Fig. 3).

### SOD and CAT activity and GSH content in the small intestine

**Figure 4:**
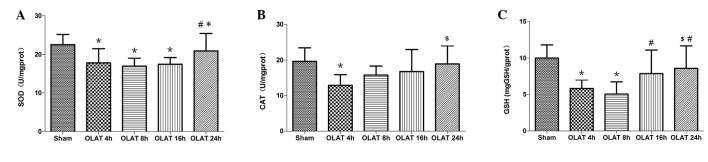
Changes of (A) superoxide dismutase (SOD), (B) catalase (CAT) activity and (C) glutathione (GSH) content in the small intestine. Results are presented as the mean ± SD (n=7/group). ^*^P<0.05 compared with the sham group; ^$^P<0.05 compared with the 4-h group; ^#^P<0.05 compared with the 8-h group; ^*^P<0.05 compared with the 16-h group. OLAT, orthotopic liver autotransplantation.

The SOD activity at 4, 8 and 16 h markedly decreased by 20–25% (P<0.05 vs. sham group). The SOD activity at 24 h gradually recovered to the sham group levels (Fig. 4).

The CAT activity greatly decreased by ~40% at 4 h following OLAT (Fig. 4; P<0.05 vs. sham group). The activity of CAT at 8, 16 and 24 h progressively recuperated the baseline levels (P>0.05 vs. sham group).

The content of GSH decreased at 4 and 8 h. The decrease was 40–50% (P<0.05 vs. sham group). The content of GSH at 16 and 24 h was higher than the content at 4 and 8 h (P<0.05; Fig. 4).

### Expression of Nrf2 in the small intestine

**Figure 5:**
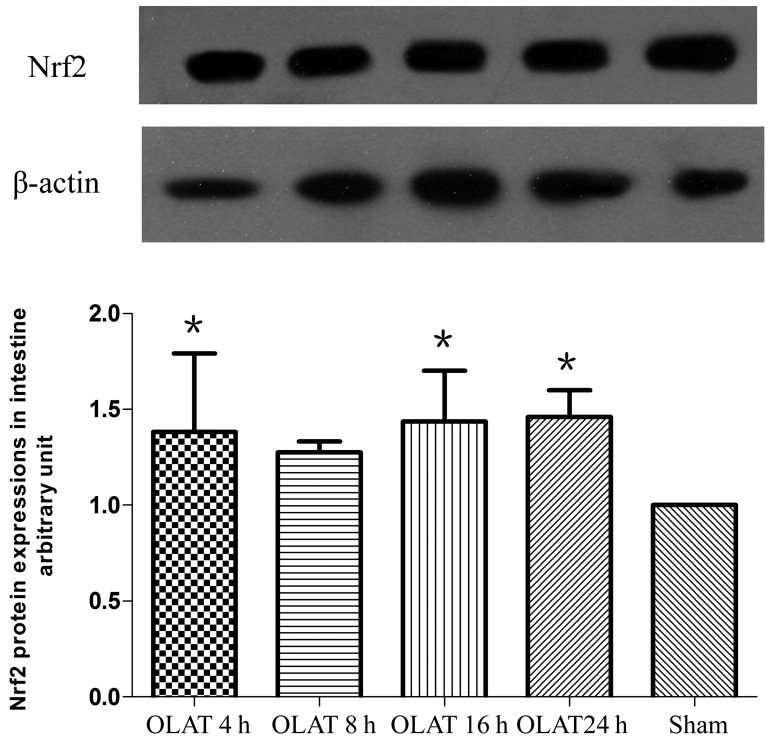
Western blot analysis of the expression of small intestine NF-E2-related factor-2 (Nrf2). Results are presented as the mean ± SD (n=7/group). ^*^P<0.05 compared with the sham group; ^$^P<0.05 compared with the 4-h group; ^#^P<0.05 compared with the 8-h group. OLAT, orthotopic liver autotransplantation.

OLAT induced a ~50% higher expression of Nrf2 (Fig. 5). Differences were observed at 4, 16 and 24 h following OLAT (P<0.05 vs. sham group).

## Discussion

Bacterial translocation and enterogenous endotoxemia following intestinal injury have been known to play a significant role in MODF and SIRS (3). Oxidative damage that is induced by ROS has a crucial role in the pathogenesis of intestinal injury. However the correlation between perioperative intestinal injury and oxidative stress in liver transplantation remains unclear. The present study used the OLAT model, which is able to simulate the key surgical procedures and pathophysiological processes that occur in liver transplantation, including hemodynamic changes, congestion, hypoxia, intestinal reperfusion subsequent to unclamping the portal vein and hepatic ischemia-reperfusion injury (17). The present study identified that OLAT causes serious injury to the pathological structure in the intestines at 8 h following the procedure. The tissues recover gradually, accompanied with an imbalance in oxidation and antioxidation. The characteristics of the intestinal damage within 24 h following OLAT were aggravated progressively and then recovered gradually.

Oxidative stress has been known to play a crucial role in organ damage, with O_2_^−^, H_2_O_2_ and ^•^OH being the major ROS (6,7). Antioxidant enzymes and antioxidants, including SOD, CAT and GSH, which constitute the defense system of the body, play a major role in scavenging oxygen free radicals, dealing with oxidative stress and maintaining the redox balance (8–10). The increased production of ROS and the decreased production or excessive consumption of antioxidant enzymes or antioxidants may cause an imbalance between oxidants and antioxidants. The MDA content may reflect the degree of lipid peroxidation damage (18). Karabulut *et al*(19) investigated the changes in the levels of ileum MDA, GSH and nitric oxide (NO) in rats that underwent 45 min of hepatic ischemia and 30 min reperfusion, and the results demonstrated that oxidative damage in the ileum was demonstrated by a significant decrease in GSH and an increase in MDA and NO levels. More recently, Tanrikulu *et al*(20) revealed that hepatic ischemia for 60 min and a reperfusion time of 90 min caused histological injury of the intestinal mucosa accompanied by an increase in intestinal MDA and a decrease in GSH-peroxidase. These studies demonstrated that the oxidant and antioxidant imbalance participates in intestinal damage induced by hepatic ischemia-reperfusion.

Hepatic ischemia-reperfusion and intestinal congestion or hypoxia-reperfusion are the main procedures of liver transplantation, but it is uncertain whether or not these procedures change the balance of intestinal oxidants and antioxidants. The present study demonstrated that all the observed oxidative indexes changed most significantly at 8 h following OLAT, showing that OLAT induced a 1.6–1.8-fold increase in H_2_O_2_ and ^•^OH production, as well as a 2–2.4-fold increase in MDA, a decrease (20–40%) in SOD and CAT activity and a significant decrease (40–50%) in GSH. The amplitude of the oxidative and antioxidative indexes decreased at 16 and 24 h following OLAT. The results of the present study indicated that the imbalance between oxidants and antioxidants contributed to intestinal oxidative injury induced by liver transplantation, particularly in the early phase, while the gradual restoration of the oxidation/antioxidation system balance was beneficial to promoting the repair of the intestine. This investigation indicates that antioxidative intervention is required to alleviate intestinal oxidative injury. Based on the results of the present study, severe complications, including SIRS or MODS following liver transplantation, may be effectively prevented.

Nrf2 is an oxidative-stress sensitive transcription factor. Following the phosphorylation of serine/threonine, stimulated by active oxygen, Nrf2 combines with antioxidative response element to form a complex that regulates the genetic expression of antioxidase, therefore protecting against oxidative injury by enhancing antioxidant capacity (11–13,21,22). Lu *et al*(23) used Nrf2-knockout mice and observed that pyrazole caused oxidative liver damage, which was severe in the Nrf2-knockout mice compared with the wild-type mice. Jin *et al*(24) revealed that Nrf2-deficient mice were more susceptible to traumatic brain injury-induced acute intestinal mucosal injury than wild-type Nrf2^+/+^ mice, as characterized by decreased intestinal mRNA expression and antioxidative and detoxifying enzyme activities, including NAD(P)H:quinone acceptor oxidoreductase 1 and glutathione *S*-transferase A1. The present study identified that the expression of intestinal Nrf2 increased at each time point following OLAT, demonstrating that ROS may promote the activation of Nrf2 and therefore initiate the process of endogenous antioxidation. The extent of alteration to all the parameters at 8 h following OLAT was further identified; Nrf2 expression was increased (~40%), the ROS level was raised >82% and the MDA levels were increased by 2.4-fold. However, the activity of the antioxidative enzymes decreased. Nrf2 did not offer enough antioxidative enzymes against the intestinal injury that was caused by ROS in the early phase following the graft. However, it may be hypothesized that the intestine may be repaired gradually with an increase of the antioxidase activation regulated by Nrf2.

In conclusion, OLAT results in intestinal damage, which is consistent with the imbalance between oxidation and antioxidation. The upregulation of Nrf2 following OLAT is not sufficient to withstand the oxidative injury in the intestine.
